# Role of Minerals and Trace Elements in Diabetes and Insulin Resistance

**DOI:** 10.3390/nu12061864

**Published:** 2020-06-23

**Authors:** Pallavi Dubey, Vikram Thakur, Munmun Chattopadhyay

**Affiliations:** 1 Department of Obstetrics and Gynecology, Texas Tech University Health Sciences Center El Paso, El Paso, TX 79905, USA; padubey@ttuhsc.edu; 2 Department of Molecular and Translational Medicine, Texas Tech University Health Sciences Center El Paso, El Paso, TX 79905, USA; vikram.thakur@ttuhsc.edu; 3 Graduate School of Biomedical Sciences, Texas Tech University Health Sciences Center El Paso, El Paso, TX 79905, USA

**Keywords:** minerals, trace elements, diabetes, insulin

## Abstract

Minerals and trace elements are micronutrients that are essential to the human body but present only in traceable amounts. Nonetheless, they exhibit well-defined biochemical functions. Deficiencies in these micronutrients are related to widespread human health problems. This review article is focused on some of these minerals and trace element deficiencies and their consequences in diabetes and insulin resistance. The levels of trace elements vary considerably among different populations, contingent on the composition of the diet. In several Asian countries, large proportions of the population are affected by a number of micronutrient deficiencies. Local differences in selenium, zinc, copper, iron, chromium and iodine in the diet occur in both developed and developing countries, largely due to malnutrition and dependence on indigenous nutrition. These overall deficiencies and, in a few cases, excess of essential trace elements may lead to imbalances in glucose homeostasis and insulin resistance. The most extensive problems affecting one billion people or more worldwide are associated with inadequate supply of a number of minerals and trace elements including iodine, selenium, zinc, calcium, chromium, cobalt, iron, boron and magnesium. This review comprises various randomized controlled trials, cohort and case-controlled studies, and observational and laboratory-based studies with substantial outcomes of micronutrient deficiencies on diabetes and insulin resistance in diverse racial inhabitants from parts of Asia, Africa, and North America. Changes in these micronutrient levels in the serum and urine of subjects may indicate the trajectory toward metabolic changes, oxidative stress and provide disease-relevant information.

## 1. Introduction

Minerals and trace elements are essential micronutrients required for the normal functioning of the body. These elements are particularly beneficial for physiological functions [1]. Minerals and trace elements are essential for many biochemical reactions, present as stabilizing components of enzymes and proteins and function as cofactors for many enzymes. Certain trace elements regulate crucial biological processes by binding to the receptor site of the cell membrane or by changing the shape of the receptor to prevent entry of particular molecules into the cell [2]. Micronutrients serve dual roles: they maintain the stabilization of the cellular structures at their optimal levels, but their inadequacy proceeds to alternate pathways and may cause ailments [3]. These essential micronutrients have important physiological implications and exhibit direct associations with diabetes mellitus [4,5].

Scientific evidence and clinical data from diabetes research are reliable sources for essential micronutrients deficiency/overload estimation. However, the many inconsistent studies make it difficult for physicians to establish nutritional recommendations for diabetics [6]. Due to progress in interventions and research, the life expectancy of diabetic subjects has risen with an overall increase in the geriatric population. Trace element associated antioxidant enzymes are altered in diabetes [7]. Many cohort studies have shown that the homeostasis of trace elements can be altered with diabetes mellitus [8]. Early imbalances in specific elements may play an important role in disrupting insulin metabolism [9,10,11]. The majority of cohort studies focus either on a single element or on a limited combination of elements only.

**Figure 1:**
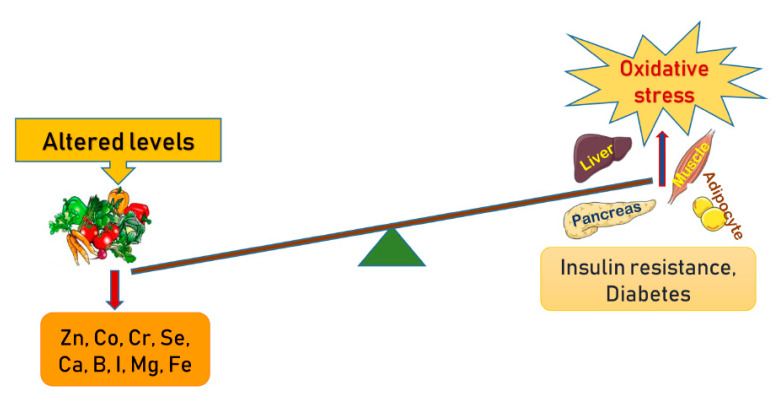
Schematic presentation of the altered levels of trace elements and minerals in the manifestation of oxidative stress in amplifying pathways towards insulin resistance and development of diabetes.

Micronutrients are identified as vital nutrients that are required in trace amounts for homeostasis, enzyme regulation and functioning [12,13]. Macro elements, vitamins, trace elements and organic acids are the four major classes of micronutrients. Macro elements primarily include chloride, calcium, phosphorous, magnesium, sodium, potassium and iron, whereas certain trace elements like cobalt, boron, chromium, copper, sulfur, iodine, zinc and molybdenum enhance insulin action by activating insulin receptor sites [14]. These trace elements play specific roles in the pathogenesis and progression of type 2 diabetes mellitus (T2DM) and the mode of action of a number of macro and trace elements is altered in T2DM [15]. This review includes various randomized controlled trials, cohort and case-controlled studies and observational and some laboratory-based studies with substantial outcomes. This comprehensive review gathers studies on diverse racial inhabitants from parts of Asia, Africa and North America. Overall this review validates that trace element deficiencies may directly or indirectly be associated with oxidative stress that ultimately precedes to insulin resistance or diabetes (Figure 1).

## 2. Boron

Boron, an important but often underused trace micronutrient found in certain foods, plays a diverse and significantly important role in metabolism [16,17]. The most relevant properties of boron in relation to human health include bone development and regeneration, wound healing, sex hormone production, vitamin D metabolism, and the absorption and use of calcium and magnesium [17,18,19]. Studies have shown that dietary boron modulates plasma insulin concentrations. Bakken et al. showed that rats deprived of boron had significantly higher plasma insulin concentrations than rats supplemented with boron. Boron deficiency demonstrated no association with corresponding changes in plasma glucose concentrations and is also not dependent on magnesium or dietary vitamin D status [20]. Boric acid inhibits Ca^2+^ release in response to ryanodine receptor agonist through binding NAD+ and/or cyclic ADP ribose and inhibiting the release of Ca^2+^, which further affects insulin release and brain function [21]. Animal studies revealed that boron affects triglyceride levels and may act as a metabolic regulator in enzymatic systems. However, a study confirmed that the maternal status of boron in normal and diabetic pregnancies is not correlated with lipids and boron levels. The serum lipids and boron levels in 15 non-gestational diabetic and 19 gestational diabetic women showed no significant change in levels of boron [22]. Another study demonstrated that boric acid and sodium pentaborate pentahydrate (NaB) showed inhibitory activities on adipogenesis in a cell model. Boron treatment repressed the expression of adipogenesis-related genes and proteins by regulating critical growth factors, β-catenin, AKT and extracellular signal-regulated kinase signaling pathways [23]. Boron treatment also demonstrated a decrease in oxidative stress in diabetic animals therefore showing an antioxidant effect with pancreatic beta-cell preservation [24].

## 3. Calcium

Calcium homeostasis plays major roles in insulin resistance and secretion [25]. Calcium homeostasis is impaired in diabetes and contributes to defective cell regulation in erythrocytes, cardiac muscles, platelets and skeletal muscles. The impaired homeostasis is concerning as it could be a significant contributory factor in the regulation of proper insulin secretion and action, also affecting various vascular complications independently [26,27].

In 2007, Pittas et al. showed that changes in calcium and vitamin D levels appear to be involved in the development of T2DM. The study showed a moderately consistent association between low vitamin D status and calcium or dairy intake, and prevalence of T2DM or metabolic syndrome. The serum 25-hydroxyvitamin D (25-OHD) levels and prevalence of metabolic syndrome and T2DM were analyzed, showing inverse associations with incidence of T2DM or metabolic syndrome for the highest versus lowest combined vitamin D and calcium intake. Hyperglycemia produced an adverse influence with vitamin D and calcium deficiency, whereas supplementation with these two nutrients showed beneficial effects on glucose metabolism [28].

In two small group studies, a contrast in the levels of serum calcium was reported. One study with 30 subjects in Baghdad with an age range of 30–70 years reported an increase in the amount of serum calcium with a substantial decrease in the parathyroid levels [29]. Another study performed in India reported significantly decreased levels of serum calcium in diabetic patients compared with the non-diabetic controls. Increased plasma blood glucose levels were negatively correlated with serum calcium levels [30]. In Khartoum, North Sudan, a cross-sectional study was performed in 40 patients with T2DM and healthy controls to evaluate serum levels of calcium and glycated hemoglobin (HbA1c). The results showed that the diabetic group with increased Hb1Ac experienced a substantial decrease in serum calcium levels compared with the control group with normal levels of HbA1c. This negative correlation between the serum calcium levels and HbA1c in diabetic patients suggests that uncontrolled hyperglycemic diabetic patients are at risk of hypocalcemia when compared with control patients [31].

Cohort studies examining the role of elevated serum calcium levels as markers of impaired glucose metabolism are lacking. One such study demonstrated an elevated risk of diabetes in individuals with increased serum calcium concentrations. The study concluded that 77 cases of T2DM showed an overall increase in serum calcium levels during follow-up. These results are in line with previous cross-sectional studies in which patients with diabetes showed higher serum calcium levels than non-diabetic individuals, which continued to be significant after individuals taking calcium supplements or having calcium levels out of the normal range were excluded, thereby demonstrating the increased risk of T2DM with increased serum calcium levels [32]. Another study confirmed the prevalence of metabolic syndrome and diabetes with higher serum calcium levels in 1329 middle-aged and elderly Korean subjects (*p* < 0.001). This association was independent of age, sex, body mass index (BMI), serum creatinine, phosphorus, parathyroid hormone (PTH), 25-OHD levels, smoking, alcohol drinking, exercise, total energy and calcium and sodium intake [33]. Studies have shown a complex association between calcium levels and the pathogenesis of diabetes. Decreased β-cells function was related to abnormal calcium regulation [34] which could further connect to altered glucose homeostasis and oxidative stress [35]. Cell culture studies showed that high cytosolic calcium levels may be associated with insulin resistance [36]. Earlier dose-dependent meta-analysis of cohort studies have shown that dietary intake of calcium prevents the development of T2D [37,38].

## 4. Cobalt

A number of studies have reported that typical serum values of cobalt are less than 0.5 µg/L. Saker et al. showed that cobalt chloride (CoCl_2_) decreased gluconeogenesis in diabetic rats through its glucose-lowering effect [39]. Cobalt alone or along with ascorbate reduces lipid peroxidation in visceral organs of diabetic rats [40]. Serum level of cobalt declined in T2D compared to non-diabetic counterparts and cobalt treatment also showed amelioration in nephropathy as well as heart function in a rat model of type 2 diabetes by alleviating oxidative stress [41].

The studies performed on human subjects to assess the levels of cobalt in diabetic patients and respective controls are inadequate. One study based in Pakistan targeted diabetic and non-diabetic men in five age groups [42]. On multi-element serum analysis, they reported a higher mean concentration of cobalt in diabetic patients, which is contradictory to the previous studies performed on streptozotocin (STZ) treated Type 1 diabetic rats. Flores et al. reported significantly higher serum concentrations of Al, Cd, Cu, Mn, Hg and Ni, and lower Cr, Co and V in diabetic patients compared to healthy subjects (unpaired *t*-test, *p* < 0.05) [43]. The levels of trace elements in the serum and urine of diabetic patients and healthy subjects were analyzed for 76 subjects in the age group 52 ± 8 years. The study confirmed higher urine levels of Cr, As, Cu and Zn and lower levels of Cd, Co, Pb, Mn, Mo, Ni and Se in diabetic patients compared to healthy subjects. However, only the differences in Cd and Zn were statistically significant [43]. An earlier study had shown that 2 mM of hexamminecobalt chloride inhibited 22.2 mM of glucose-induced insulin secretion in mouse pancreatic islets cells without impeding glucose metabolism and Ca^2+^ influx into the cytosol [44].

## 5. Chromium

Since the discovery of chromium (Cr) as an essential trace metal in 1955 [45], it has been found to effectively improve glucose tolerance by reducing insulin resistance. A China-based study showed that supplemented Cr improved the blood glucose, insulin, cholesterol, and HbA1C levels of T2DM patients in a dose-dependent manner [46]. Proper chromium nutrition improves blood lipid profile and insulin action [47]. Most diets cannot fulfill the suggested intake of 50 mg for Cr. Inadequate Cr leads to signs and symptoms similar to those of diabetes and cardiovascular diseases [48]. Chromium improves the glucose/insulin levels in subjects with hypoglycemia, hyperglycemia, diabetes and hyperlipidemia, with no detectable effects on control subjects. Chromium also improves insulin binding, receptor number and insulin receptor enzymes by increasing insulin sensitivity, β cell sensitivity and insulin internalization [49].

Three controlled studies on Cr (III) supplementation with subjects with impaired glucose tolerance showed no considerable effects [50,51,52], whereas 12 studies on Cr interventions reported improved blood lipid profile of subjects ranging from malnourished children [53,54] to healthy middle aged individuals [55,56,57,58,59,60,61]. Subsequent studies have reported that dietary Cr acts as a physiological enhancer of insulin activity and was termed a glucose tolerance factor (GTF) [62,63,64]. Kazi et al. performed a study in Pakistan, where 166 healthy and 257 diabetic subjects of both sexes, aged 45–60 and 61–75 years were recruited, and their whole blood, urine and scalp hair were collected to study a number of essential trace elements including Zn, Fe, Ni, Cu, Mn and Cr. The study reported that the blood and scalp hair samples of diabetic subjects had reduced levels of Cr, Zn and Mn compared with their control counterparts (*p* < 0.001). Higher levels of Cu and Fe were observed in the diabetic subjects than in healthy controls in scalp hair and blood, though the difference found in blood samples was not significant (*p* < 0.05) [65]. Another study on the effect of chromium supplementation on lipid levels and glucose metabolism revealed that chromium had no effect on lipid or glucose metabolism in people without diabetes, whereas it significantly improved glucose metabolism in diabetic patients [66]. Cr participates in increased insulin binding, increased insulin receptor number and increased insulin receptor phosphorylation. A comparative study performed in China and the United States revealed that mildly glucose intolerant subjects required only 200 mg/day Cr supplementation whereas people with higher glucose tolerance and diabetes required more [67]. Rajendran et al. concluded the relationship between serum Cr levels and T2DM. According to them, a decrease in Cr levels occurred as a consequence of metabolic response to oxidative stress in T2DM patients. In this study, 42 newly diagnosed T2DM patients were divided into two groups: well controlled (HbA1c ≤ 7.0%) and uncontrolled groups (HbA1c > 7.0%) and serum Cr concentration was measured. T2DM patients with uncontrolled glucose levels demonstrated lower serum Cr levels (0.065 ± 0.03 µg/L versus 0.103 ± 0.04 µg/L, *p* < 0.05) compared with the control group. The HbA1c and serum Cr levels were inversely correlated, which was statistically significant (r = –0.6514, *p* < 0.0001). Advancing age contributed to a decrease in chromium levels in both the groups after 40 years of age (*p* < 0.05) [68]. A separate study with an experimental diabetic rat model established that hyperglycemia-mediated oxidative stress was attenuated by oral chromium picolinate administration [69].

## 6. Iodine

Iodine deficiency leads to decreased thyroid hormone synthesis, which in turn leads to increased thyroid stimulating hormone (TSH) secretion and increased thyroid gland growth [70]. A recent study showed that excess of iodine diminished cell viability and compromised the function of insulin secretion in Islet β cells that could be mediated through endoplasmic reticulum stress and by inducing (pro-apoptotic) proteins [71]. Thyroid function is essential for regulating energy metabolism, and abnormal thyroid function may have substantial effects on blood glucose control in diabetes. Patients with diabetes mellitus are at an increased risk of thyroid disease [72,73]. A study was conducted to assess the clinical implication of iodine status and the levels of urinary iodine in T2DM patients. In this Riyadh cohort study, a total of 266 adults from Saudi Arabia aged 18 to 55 years (109 T2DM patients and 157 healthy controls) were randomly selected. Subjects were evaluated for fasting glucose and lipid profile, serum concentrations of TSH, T3, and T4, as well as urine creatinine and urine iodine. The study revealed lower urine iodine concentration in T2DM compared with healthy control subjects (84.6 ± 2.3 versus 119.4 ± 3.4, *p* < 0.001) [74]. Insulin resistance (IR) is a causative agent of pathogenesis of impaired glucose metabolism and is associated with increased thyroid volume and nodule prevalence in patients with metabolic syndrome [75,76]. In a study by Cooppan and Kozak, 70 patients with diabetes mellitus with hyperthyroidism were studied and they found that the incidence of diabetes improved upon the treatment of hyperthyroidism [77]. A study was conducted on 156 patients with pre-diabetes, 123 patients with T2DM and 114 subjects with normal glucose metabolism to evaluate thyroid volume and nodule prevalence in patients with pre-diabetes and T2DM in a mild-to-moderate iodine deficient area. The mean TSH level in the diabetes group was higher than in the control and pre-diabetes groups. Mean thyroid volume was higher in the pre-diabetes and diabetes groups vs. control group, suggesting that patients with impaired glucose metabolism have significantly increased thyroid volume and nodule prevalence [78].

Thyroid disorders are more common in women (30%). A retrospective chart review of an American Indian population had shown an association between diabetes and hypothyroidism. A total of 156 cases of diabetes and 25 cases of hypothyroidism were identified among American Indian individuals living in the service area from a rural isolated northeastern tribe. In women, hypothyroidism and diabetes were more prevalent (5% and 21%, respectively) than men (13% and 0.2%, respectively). Hypothyroidism was more prevalent in women aged 60 years and older (21%) compared to younger women (5%), as assessed by the overall ratio of women with diabetes (8.8%), suggesting that American Indian women have a high prevalence of coexistence of diabetes [79].

## 7. Iron

Iron affects glucose metabolism. The bidirectional association between glucose homeostasis and iron metabolism is being increasingly acknowledged [80]. Impaired iron uptake could be a contributing factor that affects glucose metabolism. Serum ferritin concentration in T2DM patients may affect insulin sensitivity, vascular resistance, viscosity, and oxidative damage. Both serum ferritin levels and BMI may act as independent predictors in a glucose tolerance test [81].

One study was performed with an oral glucose tolerance test (OGTT) in pregnant women without anemia or diabetes mellitus before 20 weeks of gestation and they were tested again at 28 ± 3 weeks of gestation to measure serum iron, ferritin and transferrin levels. The records were evaluated after delivery. Out of 401 women, 97 were diagnosed with gestational diabetes mellitus (GDM), and they were compared with the control group from the at-risk nondiabetic cases. The results revealed no change in the weight, BMI, or hemoglobin levels in the third trimester, but showed significantly higher concentrations of serum ferritin, iron, transferrin saturation and postnatal hemoglobin, suggesting a correlation between increased iron stores and glucose intolerance [82].

The increased risk of anemia in diabetic patients was studied in a survey where 820 subjects with diabetes were examined in a long-term follow up. A number of tests, including blood test, urine test and a full blood count were performed on these patients over a period of two years. Almost 23% of patients had unrecognized anemia, which was two to three times higher than in patients with renal impairment. The increased risk for anemia in patients with diabetes could be a consequence of other micronutrient deficiencies [83]. In this cross-sectional study, plasma iron indices were assessed, which showed increased transferrin saturation (>35%) in diabetic patients that was three-to-four-fold higher compared with non-diabetic counterparts. This study also demonstrated that increased transferrin saturation levels are associated with low C-reactive protein and elevated fasting plasma glucose levels, which was more frequent in male patients (*p* < 0.0001). No possible association was found between the presence of elevated iron indices in the T1DM patients with presence of diabetic complications [84]. In a prospective nested case-control study, plasma ferritin level was surveyed and ferritin to transferrin receptors ratio was estimated in relation to the possibility of development of T2DM. At the time of recruitment during 1989–1990, the 32,826 women subjects who were chosen did not have diabetes, cardiovascular disease or cancer. The blood test results at 10 years in a follow-up study showed that 698 women developed diabetes, with 716 BMI, age, fasting status and race-matched controls examined and compared. Increased ferritin levels were correlated with an increased risk of T2DM in healthy women (independent of known risk factors for diabetes), which was further associated with a lower ratio of transferrin receptors to ferritin [85]. 

Iron is a strong pro-oxidant and the association of increased level of oxidative stress with high body iron levels can increase the risk of T2DM [86]. Many epidemiological studies have described a link between high body iron stores via circulating ferritin levels with T2D and of other insulin resistant states [87]. Phlebotomy improved insulin sensitivity in humans by reducing body iron levels, which suggests a link between iron overload and diabetes risk [88]. Iron is abundant in the placenta and contributes to the production of free radicals, which play a role in GDM, affecting about 7% of all pregnancies. To compare iron status at 24–28 weeks of pregnancy, 34 women diagnosed with GDM and 34 non-GDM women in the control group were tested for ferritin, hemoglobin (Hb), serum iron, mean corpuscular volume (MCV), total iron-binding capacity (TIBC) and mean corpuscular hemoglobin (MCH). The tests revealed that TIBC was lower in the GDM group, whereas the levels of serum ferritin, MCV, MCH iron, transferrin saturation and hemoglobin were higher in the GDM group [89]. The incidence and risk of anemia were assessed according to sex and glycemic control status in 200 patients with T2DM divided into groups according to glycemic control and sex. There was a higher incidence and risk of anemia in women (36%) compared with men (27%) with controlled diabetes, indicating that poor glycemic control and sex differences are associated with the incidence of anemia in T2DM [90].

Iron plays a major role in the adscription of T2DM and not many markers of iron metabolism are known except for ferritin. Transferrin, soluble transferrin receptor (sTfR), transferrin saturation (TSAT), sTfR-to-log10ferritin (sTfR-F) index and iron with impaired glucose metabolism/prediabetics, T2DM and insulin traits were investigated in 2893 participants in a population-based cooperative health research in the region of Augsburg (KORA) F4 study in Germany. TSAT and iron were inversely associated with T2DM, thereby indicating the association of secondary iron metabolic markers in the progression of T2DM [91]. The possible influence of iron on insulin action and secretion through changes in relative excess iron was studied in 2015 and iron was found to increasingly influence glucose metabolism on multiple levels [80]. 

## 8. Magnesium

Magnesium is a cofactor required for movement of glucose into the cell and for carbohydrate metabolism. It is involved in the cellular activity of insulin. Low magnesium intake is a risk factor for diabetes [92]. Deficiency in magnesium inhibits cellular defenses against oxidation damage, which in turn results in a decreased resilience to the oxidative stress caused by diabetes, thereby expediting the progression to diabetes-related complications. Therefore, hypomagnesemia may exacerbate T2DM, but studies have also shown that magnesium intake reduces the risk of T2DM and metabolic syndrome by alleviation of insulin resistance [93,94,95]. Animal studies have shown that dietary Mg administration (50 mg/mL in drinking water) for 6 weeks decreased blood glucose levels, improved mitochondrial function and reduced oxidative stress in diabetic mice [96].

A number of studies have shown that hypomagnesemia is a common attribute of diabetes mellitus and occurs at an incidence of 13.5% to 47.7% among patients with T2DM [97,98]. In one study, the effect of insulin resistance on magnesium accumulation in erythrocytes was tested. Out of 15 Caucasians and 14 Pima Indians, the latter were more insulin resistant than the former (*p* ≤ 0.0001). A baseline fasting magnesium level in erythrocytes showed that the concentration was the same in both groups. Magnesium level was recorded again after insulin infusion, and its concentration increased less in the Pima Indians than in the Caucasians (*p* < 0.03). When the levels were adjusted for the magnitude of response to insulin, the increase was similar. The authors of this study concluded that erythrocyte magnesium accumulation is due to the degree of insulin resistance [99]. Magnesium is strongly associated with diabetes and hypertension. Cytosolic free Mg is frequently low in diabetic patients [100]. Magnesium deficiency aggravates insulin resistance. Therefore, diabetic subjects are at a higher risk of cardiovascular diseases [101], whereas the atherosclerosis risk in communities study (ARIC) found that low dietary Mg intake did not increase the risk of diabetes in a middle-aged population [102,103,104].

Magnesium is a cofactor for the downstream actions of the insulin cascade. Decreased intracellular magnesium damages tyrosine kinase activity and blocks insulin action within the cell, resulting in growing insulin tolerance. Dietary deficiency of magnesium may also be a risk factor of diabetes. A number of clinical trials found benefits from magnesium supplementation in diabetics [105].

In a cross-sectional study, the plasma levels of magnesium of diabetics were investigated. Eleven T1DM, 25 T2DM and 34 controls subjects matched for age and sex were enrolled. Atomic absorption spectroscopy was used to determine magnesium levels. Plasma magnesium concentrations were lower in both T2DM groups (*p* < 0.0001). This abnormality was easily attributed to the increased glycation of hemoglobin. The authors concluded that the impaired metabolism may play a role in the occurrence of diabetic complications [106]. Sales et al. investigated the association between magnesium levels and fasting blood glucose. Urine magnesium, plasma magnesium and erythrocyte magnesium concentrations were measured by atomic absorption spectroscopy. Subjects with T2DM were enrolled and the study measured urine, plasma and erythrocytes magnesium levels. Urine magnesium and plasma magnesium were correlated with fasting glucose levels, whereas creatinine clearance was only correlated with plasma magnesium. The authors concluded that magnesium plays an important role in maintaining blood glucose levels [107].

Malondialdehyde (MDA) and trace elements are significant factors acting as cofactors in the pathogenesis and pathophysiology of diabetes mellitus [108]. To evaluate their contribution as markers of glycemic control and lipid status in diabetic patients, trace elements Zn, Cu, Mg, Cr, Se and MDA were analyzed in a small group of 50 patients with T2DM. The patients were further divided into controlled diabetic and non-controlled (i.e., neuropathy, retinopathy) subjects. The mean levels of Zn, Mg and Se were lower in the diabetic groups, whereas MDA was higher in the diabetic groups and showed a significant positive correlation with HbA1c, cholesterol, triglycerides, low-density lipoprotein (LDL) and copper, and a negative correlation with high-density lipoprotein (HDL), zinc, magnesium and selenium [109].

## 9. Selenium

Dietary selenium (Se) is a micronutrient that is essential for the synthesis of selenoproteins to carry out biological functions. Selenoprotein is recognized for its antioxidant and cytoprotective properties, and Se supplementation is considered to be preventative due to its counteractive oxidative properties for the onset of metabolic diseases, such as T2DM [110].

In one cross-sectional analysis on U.S. adults, serum Se was measured by atomic absorption spectroscopy in a diabetic population with a fasting plasma glucose level of 126 mg/dL, diabetics with insulin treatment and a control group. After adjusting for age, sex, race and BMI, the mean difference in serum Se between diabetics and controls was 2.1 ng/mL (95% CI 0.4–0.8, *p* = 0.02). The study concluded that high serum Se is positively correlated with the prevalence of diabetes. However, no recommendation was provided about Se supplementation or restriction for diabetes prevention [111,112]. Conversely, Se treatment at 0.2 µmol/µL in drinking water of non-obese diabetic mice for 3 weeks exhibited low serum glucose levels and improved lipid metabolism [113].

Another cross-sectional study with 5423 middle-aged and elderly Chinese adults was performed to examine the correlation between diabetes and dietary selenium. The prevalence of diabetes in the study population was 9.7% [110]. A substantial positive correlation between dietary selenium intake and diabetes was indicated from the outcome of this study, which was in agreement with the conclusions of similar studies. Another cross-sectional analysis with 8876 adults over 20 years of age in the U.S. under the National Health and Nutrition Examination Survey showed a positive correlation of higher serum selenium levels and the prevalence of diabetes [111,114,115].

Selenium is essential for glutathione peroxidase (GPx) enzyme activity. Diabetes causes an increased oxidative burden due to the production of reactive oxygen species (ROS). GPx is an important cellular defense against free radicals. In one cross-sectional analysis, GPx activity and selenium levels in the blood were measured in a study population of 50 diabetics in India and China and an age matched control population of 50 healthy college students. A significant decrease was observed in both GPx activity and selenium levels in diabetic patients. The decreased activity of GPx was attributed to the decreased selenium levels. The pathogenesis of diabetes may therefore be due to oxidative stress impairment [116,117].

To study the long-term effect of selenium exposure and the risk for the onset of T2DM in healthy subjects, Mozaffarian et al. investigated the association between toenail Se and incidence of T2DM. A little over 7000 women and men participated in a follow-up study over two decades and the study found that people with the highest levels of selenium in their toenails had a 24% lower risk of developing diabetes compared with those with the lower levels of Se [118]. Conversely, in Northern Taiwan, a hospital-based case-control study of 847 adults (diabetes: non-diabetes = 1:2) over 40 years old showed that higher serum selenium levels were positively associated with increased risk of diabetes [119]. The recommended daily requirement of selenium is 55 mg for adults, which can be achieved from diet alone. Consequently, it is challenging to conclude whether there is an association between levels of selenium and the increased risk of diabetes as this may further vary between geographical locations and the source of the minerals. Hence, Se supplementation is only required in areas where high-selenium soil is scarce. 

## 10. Zinc (Zn)

Zinc is an essential micronutrient for metabolism that regulates more than 100 enzymes for protein folding, gene expression, as well as in the production and neutralization of ROS. Zn plays an important role in cell signaling and cellular processes such as cell division and apoptosis, and disturbances in zinc homeostasis are associated with diabetes and insulin resistance. Zinc reduces cytokine production and its deficiency may cause impairment of immunological functions [120]. Zinc partially functions as an antioxidant and Zn supplementation results in reduction in ROS production, which is beneficial in ageing and diabetes mellitus [121]. Zn is vital for the appropriate processing, storage, secretion and action of insulin in mammalian pancreatic cells [122], and Zn deficiency augments the cytokine-induced damage in the autoimmune attack, resulting in the destruction of the islet cell in T1DM [122]. It might contribute toward diabetes progression through genetic polymorphisms in the zinc transporter 8 gene and in metallothionein (MT)-encoding genes that are associated with T2DM [123,124]. Diabetes, insulin and zinc have a complex correlation. Diabetes affects zinc homeostasis and it is also responsible for increased urinary loss and decreases in total body zinc [125]. In 2003, a study compared zinc levels and HbA1c between children with T1DM and healthy controls. Serum zinc levels were significantly lower in diabetic children than in healthy controls. The serum zinc level correlated negatively with HbA1c. The study concluded that T1DM children were deficient in zinc and that pharmacotherapy with zinc supplementation could be a possible treatment [126]. Previous animal studies with zinc chloride supplementation (5 mg/kg) for 1 month in diabetic rats showed altered serum glucose concentration and oxidative damage markers [127].

Another study was conducted to investigate the association of serum zinc levels between non-insulin-dependent diabetes mellitus (NIDDM) and insulin-dependent diabetes mellitus (IDDM) subjects. Zinc levels were determined spectrophotometrically, which were significantly decreased in diabetic patients compared with the control population. The effect was more pronounced in NIDDM subjects compared with IDDM subjects (*p* < 0.001). This study concluded that the oxidative stress is higher in diabetes mellitus and more so in NIDDM [128]. In an exploratory analysis, the correlation between zinc serum levels and diseases such as diabetes and hypertension was explored. The study found no relationship between serum zinc levels and glucose levels. Serum zinc levels were higher in both hypertensive subjects and hypertensive subjects with diabetes. The authors concluded that the contrasting serum zinc levels between the two diseases imply different mechanisms of etiology [129]. One cross-sectional study investigated the relationship between the serum zinc levels and T1DM in children. A total of 30 T1DM children from ages 6 to 18 years were matched with 30 healthy control subjects by age and sex. Serum zinc levels were statistically insignificant (*p* = 0.4) between these two groups. No correlation was found between serum zinc levels, fasting blood sugar, severity of disease (HbA1c) and duration of the disease. The authors concluded that the serum zinc levels are independent of glycemic control [130].

In a separate study, serum and urine zinc levels were measured in pre-diabetics and diabetics from Northeast China. A total of 25 T1DM, 137 T2DM and 50 age- and sex-matched controls were enrolled. Serum zinc levels were lower in both T1DM and T2DM and urinary zinc levels were elevated in both T1DM and T2DM. Simvastatin treatment was found to be ineffective when serum and urine zinc levels were measured. The authors concluded that more research is needed on the impact of serum zinc levels on diabetes and complications of diabetes [131].

T2DM patients are prone to microangiopathic complications such as diabetic nephropathy and diabetic retinopathy. One study investigated the effect of microangiopathic complications on serum zinc levels in T2DM patients. The authors enrolled 50 T2DM patients with less than two microangiopathic complications and 50 T2DM patients with at least two microangiopathic complications in the study. Serum zinc concentration was significantly lower in patients with at least two microangiopathic complications (*p* < 0.05). The authors attributed this finding to the adverse effects of high glucose concentration leading to a decrease in the reabsorption of zinc in the kidneys [132]. In a comparative analysis of serum zinc levels, the effect of T2DM on serum zinc levels was compared by enrollment of 50 T2DM patients and 50 healthy controls. Individuals with hypertension, renal disease, and obesity were excluded from the study. T2DM patients had significantly lower serum zinc levels compared with the control subjects (*p* < 0.0001). The authors concluded that diabetes disrupts Zn metabolism [133]. One study compared the serum levels of zinc between T2DM and healthy controls, enrolling 50 T2DM and 25 control subjects in the study. They found that serum zinc levels were significantly lower in diabetic patients (*p* < 0.05). No correlation was found between trace serum elements like zinc and lipid profiles. The authors concluded that impaired metabolism of elements such as zinc could play a role in the pathogenesis of diabetics [134].

## 11. Conclusions

Diabetes mellitus can alter the concentrations of trace elements, which may further lead to changes in the nutritional status of an individual. Although some micronutrients are known to be involved in the pathogenesis and progression of diabetes mellitus, others may only be a consequence of depleted or altered carbohydrate intolerance and insulin resistance. The studies often report contradictory results. The serum or tissue contents of certain elements, such as copper, manganese, iron and selenium, may be higher in diabetic patients than in non-diabetic controls. Although the majority of diabetic patients do not have micronutrient deficiencies, zinc, chromium, and magnesium deficiencies have been identified in a subgroup of patients. More cohort studies are necessary to identify the micronutrient deficiencies in diabetes mellitus. This review demonstrates that trace element deficiencies—either directly or indirectly—are associated with oxidative stress which eventually leads to insulin resistance or diabetes (Table 1).

## Figures and Tables

**Table 1:**
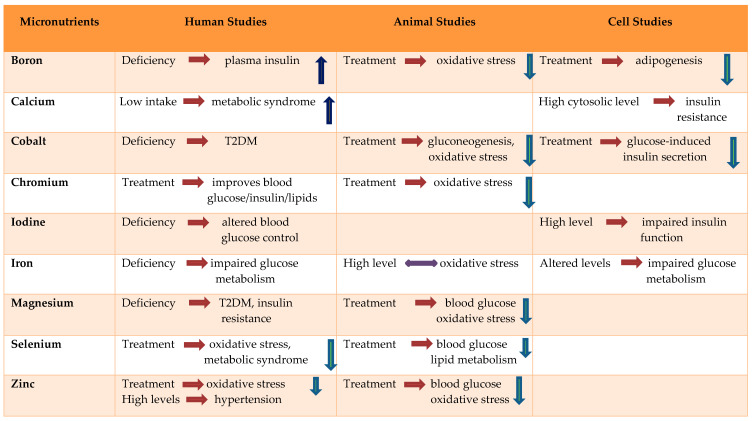
Effect of trace elements and minerals in diabetes, insulin resistance and oxidative stress.
